# The complete chloroplast genome sequences of an endemic species of Urticaceae (*Debregeasia hekouensis*)

**DOI:** 10.1080/23802359.2021.1993761

**Published:** 2021-10-23

**Authors:** Zhao-Yang Sun, Jie Liu, Moses C. Wambulwa, Zhong-Hu Li, Zeng-Yuan Wu

**Affiliations:** a Key Laboratory of Resource Biology and Biotechnology in Western China, Ministry of Education, College of Life Sciences, Northwest University, Xi’an, China; b Germplasm Bank of Wild Species, Kunming Institute of Botany, Chinese Academy of Sciences, Kunming, China; c CAS Key Laboratory for Plant Diversity and Biogeography of East Asia, Kunming Institute of Botany, Chinese Academy of Sciences, Kunming, China; d Department of Life Sciences, South Eastern Kenya University, Kitui, Kenya

**Keywords:** *Debregeasia hekouensis*, complete chloroplast genome, phylogenetic analysis, Urticaeceae

## Abstract

*Debregeasia hekouensis,* which belongs to the nettle family (Urticaceae), is a local endemic species in Hekou County, Yunnan Province, China. To provide a basis for the development of effective molecular markers for its conservation, we sequenced the chloroplast (cp) genome of *D. hekouensis* in the present study. The total length of the chloroplast(cp) genome was 155,941 bp, and exhibited a typical quadripartite structure, with a pair of IRs (inverted repeats; 25,664 bp in length) being separated by a small single copy (SSC) region of 19,085 bp and a large single copy (LSC) region of 85,528 bp. The cp genome contained a total of 112 genes, including 78 protein-coding genes, 30 tRNA genes, and 4 rRNA genes. The GC content of the entire cp genome, LSC region, SSC region, and IR region was 36.3%, 34.0%, 29.4%, and 42.7%, respectively. Phylogenetic analysis indicated that *D. hekouensis* is evolutionarily closer to *Debregeasia orientalis* and *Debregeasia squamata*.

*Debregeasia* Gaudich. comprises eight species (*D. longifolia*, *D. elliptica*, *D. orientalis*, *D. saeneb*, *D. squamata*, *D. wallichiana*, *D. australis*, and *D. hekouensis*) with a worldwide distribution, but mainly found in subtropical or tropical areas of eastern Asia (Chen et al. 2003; Wilmot-Dear and Friis 2012; Wu et al. 2018). *Debregeasia hekouensis*, first described in 2016 (Wang 2016), is an endemic species narrowly distributed in the fragmented tropical rain forest along the Nanxi River of Hekou County, Yunnan Province, China. Although phylogenetic (Wu et al. 2013, 2018) and phylogenomic (Wang et al. 2020) relationships within *Debregeasia* have been tested in previous studies, *D. hekouensis* was not included. In the current study, we reported the complete chloroplast genome (cpDNA) sequence of *D. hekouensis,* and explored its phylogenetic relationships with related species in Rosales. Our results will significantly contribute to the molecular systematics and conservation genetics of this species.

Fresh, young, and clean leaf materials of *D. hekouensis* were collected from Nanxi Town, Hekou County, Honghe Prefecture, Yunnan Province, China (22.67°N; 103.94°E). Voucher specimens (No. HKD03) were deposited at the herbarium of the Kunming Institute of Botany, China (KUN) (contact person: Zeng-Yuan Wu, wuzengyuan@mail.kib.ac.cn). Total genomic DNA was extracted using the CTAB method (Doyle and Doyle 1987). Library construction was performed with NEBNext Ultra II DNA Library Prep Kit for Illumina (New England BioLabs) following the manufacturer’s instructions. Sequencing was implemented using the Illumina HiSeq X Ten platform, which produced 150 bp paired-end reads. The expected sequencing quantity was ca. 4 Gigabyte. The raw reads were filtered to obtain clean reads, which were subsequently, assembled into circular contigs using GetOrganelle toolkit (Jin et al. 2020). Finally, the cpDNA was annotated using geneious v.9.0.2 software package (https://www.geneious.com/). The reference genomes used for assembly and annotation are the complete chloroplast genome of *D. orientalis* (GenBank accession: NC_041413).

Raw reads of *D. hekouensis* were deposited in the NCBI Sequence Read Archive (SRA: SRX10910957) and the final annotated chloroplast genome sequence was deposited in NCBI GenBank (accession number: MZ159965). The total length of the chloroplast genome was 155,941 bp, and had a typical quadripartite structure, in which a pair of IRs (inverted repeats) of 25,664 bp was separated by a small single copy (SSC) region of 19,085 bp and a large single copy (LSC) region of 85,528 bp. The GC content of the entire cp genome, LSC region, SSC region, and IR region was 36.3%, 34.0%, 29.4%, and 42.7%, respectively. The assembled cp genome contained a total of 112 genes, including 78 protein-coding genes, 30 tRNA genes, and 4 rRNA genes. Of these, a total of 17 genes were duplicated in the inverted repeat regions, 16 contained one intron each, while three (ycf3, rps12, and clpP) had two introns each.

A total of 18 cp genome sequences of Rosales species were downloaded from the NCBI database, and subjected to phylogenetic analysis. After using MAFFT for aligning (Katoh and Standley 2013), the ModelFinder (Subha et al. 2017) within the IQTREE (Nguyen et al. 2015) was used to determine the best-fitting model for Maximum likelihood (ML) analysis. The bootstrap value based on 1,000 replicates for constructing the phylogenetic tree. Based on the analysis of the 18 species, our results showed that *D. orientalis* and *D. squamata* are the closest relatives of *D. hekouensis* (Figure 1).

The newly reported chloroplast genome here would be beneficial to developing molecular markers in *Debregeasia*, which, in turn, will lay a foundation for conservation genetic studies of this species.

**Figure 1.**
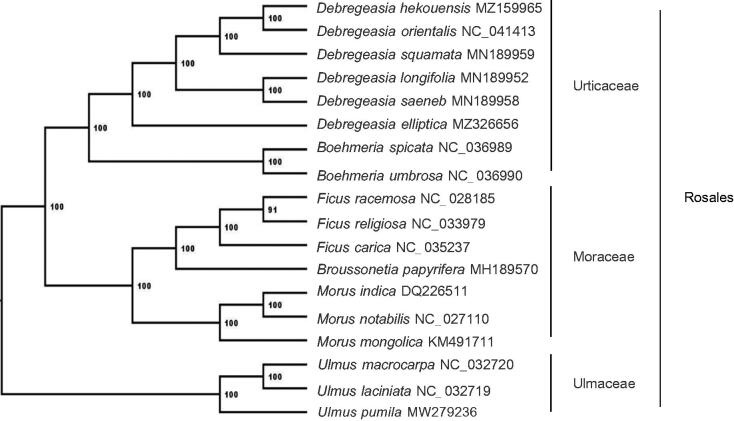
Maximum Likelihood (ML) tree of 18 species within Rosales based on the complete chloroplast genome sequences. Bootstrap support values are shown next to the nodes.

## Data Availability

The chloroplast genome sequences data of *D. hekouensis* that support the findings of this study are deposited in GenBank at https://www.ncbi.nlm.nih.gov/ under the accession no. MZ159965. The associated BioProject, SRA, and Bio-Sample numbers of *D. hekouensis* are PRJNA730369, SRX10910957, and SAMN19229652, respectively.
